# Spectral insights into the transformation and distribution of CdSe quantum dots in microorganisms during food-chain transport

**DOI:** 10.1038/s41598-017-04694-6

**Published:** 2017-06-29

**Authors:** Li-Jiao Tian, Yong Peng, Dong-Liang Chen, Jing-Yuan Ma, Han-Qing Yu, Wen-Wei Li

**Affiliations:** 1 CAS Key Laboratory of Urban Pollutant Conversion, Department of Chemistry, University of Science and Technology of China, Hefei, 230026 China; 2 Beijing Synchrotron Radiation Laboratory, Institute of High Energy Physics, Chinese Academy of Sciences, Beijing, 100049 China; 3 Shanghai Synchrotron Radiation Facility, Shanghai Institute of Applied Physics, Chinese Academy of Sciences, Shanghai, 201204 China

## Abstract

The discharge of engineered nanomaterials (ENMs) into environment is raising widespread concern not only due to their direction bio-toxicity but also their bio-concentration and bio-magnification through food web. However, the transformation and distribution of ENMs during food-chain transport are poorly understood, due to lack of accurate, reliable analytical methods. In this study, by using a suite of advanced spectrum techniques, we successfully tracked the distribution and biotransformation dynamics of CdSe quantum dots (QDs) during their transport from *Shewanella onedensis* to *Caenorchabditis elegans* in predation. Fluorescence microscopy and Raman mapping showed that the ingested QDs by *C. elegans* were located at the gut lumen and subcutaneous tissue, and were partially excreted from the nematode body over time. Micro-X-ray fluorescence (*μ*-XRF) spectroscopy and Se K-edge X-ray absorption fine structure (XAFS) results further revealed the changed distribution of Se element over time, and a shift in the major Se species from CdSe to Se^0^ and Na_2_SeO_3_^IV^. This work demonstrates the utility of advanced spectral techniques for characterizing QDs *in vivo*, and may facilitate a better understanding on the environmental transformation and fates of ENMs.

## Introduction

The growing application and environmental discharge of engineered nanomaterials (ENMs) today are posing high risks to the ecological system and human health. One major concern is that the ENMs can be transferred from low to high trophic levels through food chain. For example, polyvinylpyrrolidone-coated silver nanoparticles were found to be transferred and biomagnified through the *Escherichia coli* (*E. coli*) - *C. elegans* food chain^1^, or from green algae to grazers^2^, causing severe toxicity to the higher trophic level. The transfer of quantum dots (QDs) from ciliated protozoans to rotifers through dietary uptake^3^ and from bacteria (*Pseudomonas aeruginosa*) to ciliate (*Tetrahymena thermophila*) has also been demonstrated^4, 5^. Fluorescent QDs were favorably used in these studies because of their widespread use^6–8^ and easy traceability *in-vivo* by optical or transmission electron microscopy. The distribution, accumulation and toxicity of nanoparticles in specific living organisms have been intensively studied by using bacteria, mammalian cells and *Caenorchabditis elegans* as the models^9–11^. However, the possible changes of these ENMs during their food-chain transport were largely unknown, making their specific environmental behaviors, fate and toxicity poorly understood.

Characterization of EMNs in living organisms is challenging, due to the interference of soluble elements and complicated biological environment. The transformation between nanoparticles and their ionic forms may also occur, impeding an accurate quantification of EMNs *in vivo* ^9^. Especially, for fluorescent QDs, fluorescence quenching, emission spectrum red shift or blue shift frequently occur in conventional fluorescence microscopic observation^9^, making their quantification and localization difficult. Recently, X-ray absorption fine structure (XAFS) and micro-X-ray fluorescence (*μ*-XRF) spectroscopy was used to reveal detailed information about the chemical composition and distribution of elements or compounds *in vivo* at high spatial resolution^12, 13^. Raman mapping offers another powerful tool to visualize the molecular composition of compounds *in vivo* at subcellular scale without labeling^14^. While these methodologies have been proven useful for characterization of metallic nanoparticles^15^, here we for the first time used them to track the distribution and biotransformation of QDs during food-chain transport.

In this work, we used *Shewanella onedensis* as a representative of environmental bacteria and *C. elegans* as a representative of metazoan to constitute a simplified freshwater food chain, through which the transport and dynamic changes of CdSe QDs were investigated. *C. elegans* is broadly distributed in soil and water ecosystems and has been widely employed as a toxicology test model^16^. Since the uptake of nanoparticles by bacteria have been intensively reported and well known^17, 18^, here we focus on the downstream step, i.e., how the QDs are transported with the *S. onedensis* cells into *C. elegans*, To this end, we used *in-vivo* synthesized QDs by *S. onedensis* for simplification, *In-situ* fluorescence microscopy and Raman mapping were used to monitor the QDs distribution dynamics based on the fluorescence and chemical bond properties of QDs, respectively. The *μ*-XRF spectroscopy was used to localize the Se element distribution. The food footprints were also recorded by XAFS analysis. Together, the results reveal valuable information about the biotransformation and distribution of CdSe QDs during their food-chain transport.

## Results and Discussion

### Characterization of CdSe QDs in *S. onedensis* MR-1 cells

**Figure 1:**
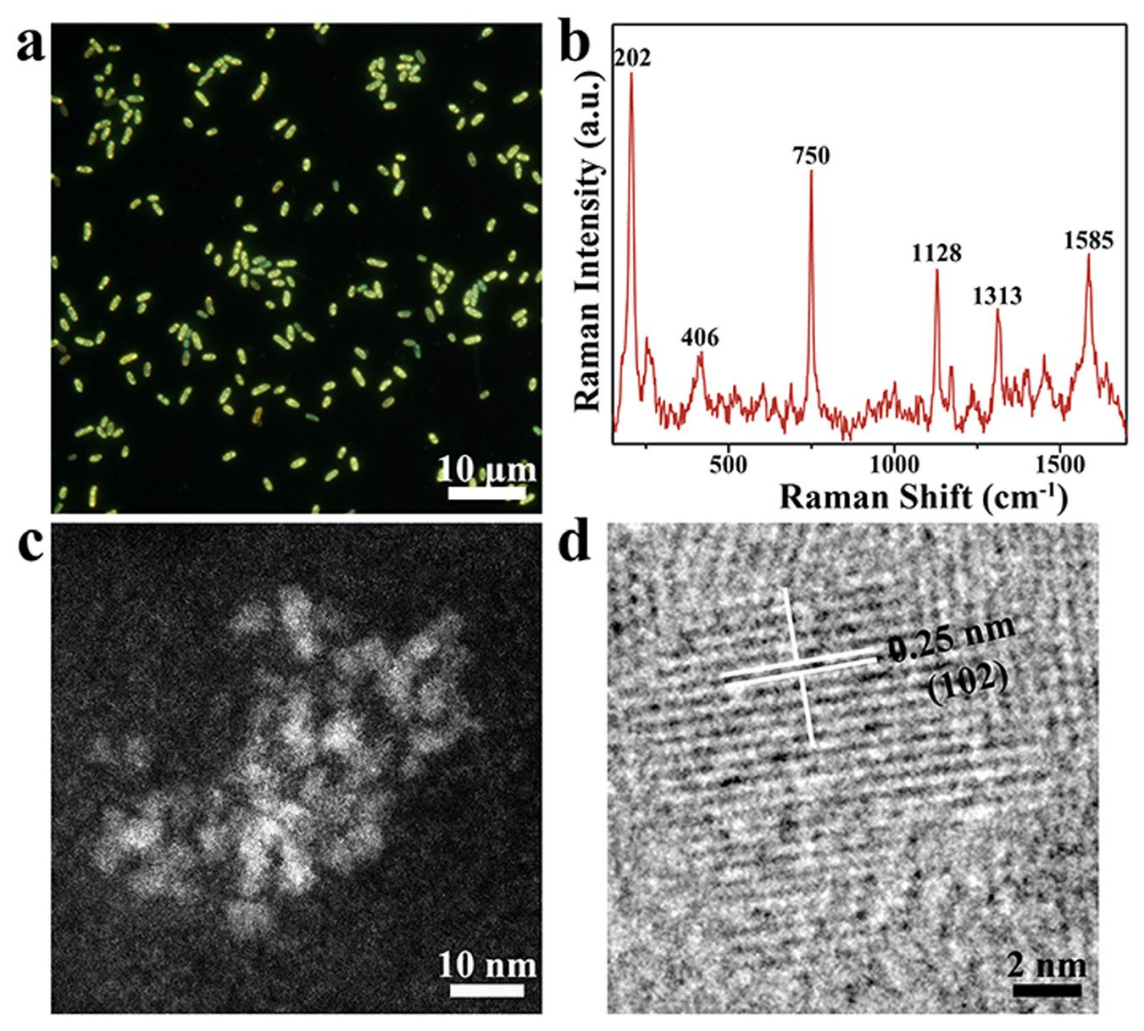
Characteristics of CdSe QDs in *Shewanella onedensis* cells. (**a**) Fluorescence image. (**b**) *In*-*situ* Raman spectrum (excited at 532 nm) shows characteristic peaks of QDs and cytochrome c. (**c**) HAADF-STEM and (**d**) HRTEM images of the purified CdSe QDs.

The fluorescent CdSe QDs in *S. ondensis* MR-1 were clearly observed by fluorescence microscopy (Fig. 1a). All the cells were lightened with bright yellow fluorescence ascribed to the *in-vivo* formed CdSe QDs. The in-situ Raman spectra of the QDs-containing *S. ondensis* cells showed several distinct peaks at 202 cm^−1^, 406 cm^−1^, 750 cm^−1^, 1128 cm^−1^, 1313 cm^−1^ and 1585 cm^−1^ (Fig. 1b). Among these, the peaks at 202 cm^−1^ and 406 cm^−1^ could be ascribed to the longitudinal-optical (LO) and 2 longitudinal-optical (2LO) phonons of CdSe, respectively^19^. The Raman characteristics of CdSe QDs were size-dependent^20, 21^, Thus, based on the Raman spectra, the average size of QDs was estimated as slightly larger than 3 nm. The Raman spectra also showed the characteristic peaks of ferrous and ferric state cytochrome C (750 cm^−1^, 1128 cm^−1^, 1313 cm^−1^ and 1585 cm^−1^) from *S. onedensis* ^22^. A direct observation of the purified QDs from *S. ondensis* cells by High-angle annular dark-field scanning transmission electron microscope (HAADF-STEM) confirmed the presence of the discrete nanoparticles with an average diameter of 3.3 nm. High-resolution TEM (HRTEM) analysis further revealed that the QDs had continuous lattice fringes with an interplanar lattice distance of 0.25 nm, corresponding to the (102) interplanar spacing of wurtzite structured CdSe.

### Distribution and movement of CdSe QDs in *C. elegans*

**Figure 2:**
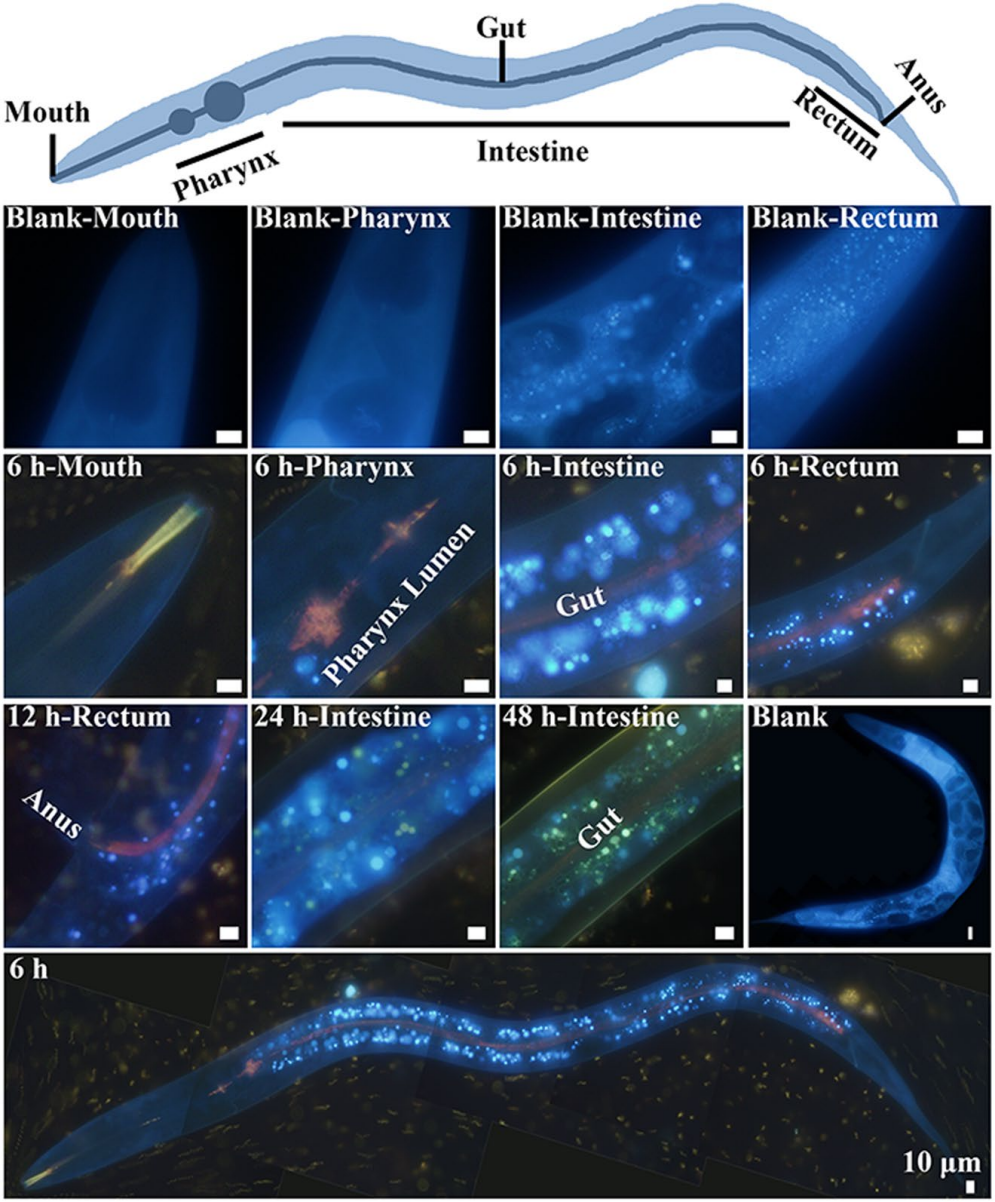
Distribution dynamics of CdSe QDs in *C. elegans* over time recorded using fluorescence microscopy. The blue signals represent the autofluorescence of *C*. *elegans*. The bright blue-green and light red fluorescence signals represent the dispersed and aggregated CdSe QDs, respectively. All the scale bars are 10 μm.

Fluorescence microscopy was used to track the distribution and movement of QD in *C. elegans* over time. According to the fluorescence images in Fig. 2, the negative control emitted blue autofluorescence only, while the experimental group showed numerous dots of bright fluorescence on the background of faint blue autofluorescence. Apparently, these bright fluorescent dots should be ascribed to the ingested QDs together with the *S. onedensis* cells. Here, the fluorescent color of QDs was distinct from the typical yellow green fluorescence of CdSe QDs because the QDs were located inside the nematode body that gave blue autofluorescence to interfere the observation. A further examination on the fluorescence dynamics showed that the cell-embodied QDs were ingested from the mouth, through the pharynx lumen, gut and finally to the rectum of the *C. elegans*. In this process, the fluorescence color changed from yellow to light red, likely attributed to the aggregation of the QDs in the acidic microenvironment of pharynx lumen^23^. A closer observation showed that, after 6-h exposure to the QDs-containing *S. onedensis* cells, the aggregated QDs (in red color) were abundantly present in the gut lumen and some dispersed QDs (in blue-green color) also entered the adjacent intestinal cells. The color change indicates that the QDs shifted back to the original state when transported from the gut lumen to adjacent intestinal cells. After 12-h exposure, the QDs became more enriched in the gut lumen and a part of the QDs were excreted from the anus of nematode. A comparison of the intestinal fluorescence signals at different time points clearly showed an expansion of the lightened area from the intestinal tract to other subcutaneous tissue and at the same time the fading of fluorescence in the gut lumen, further confirming the movement of QDs from gut to subcutaneous tissue and excretion.

### Co-localization of QDs and *S. onedensis* in *C. elegans*

**Figure 3:**
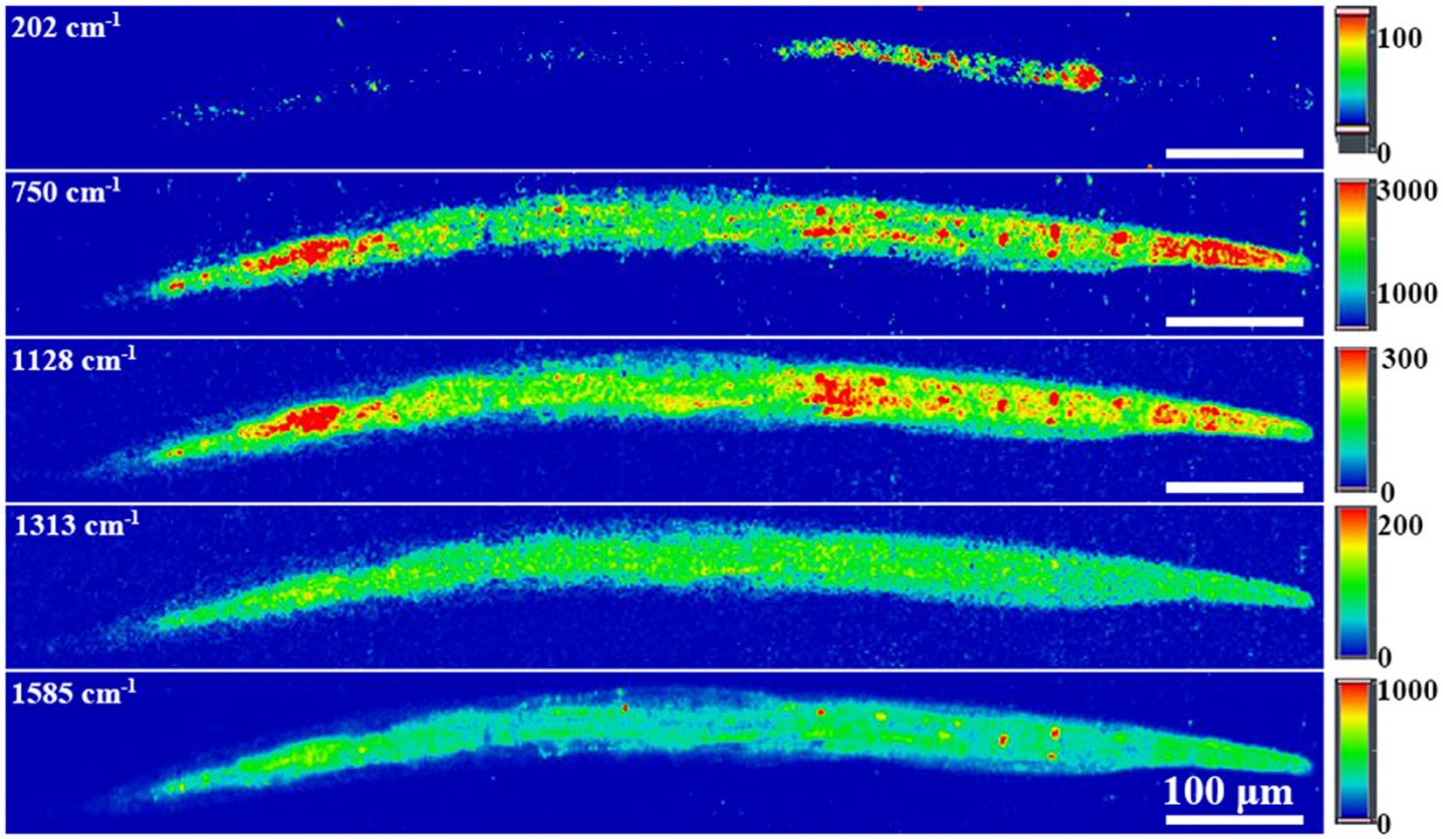
Raman mapping of CdSe QDs (202 cm^−1^) and cytochrome C (750 cm^−1^, 1128 cm^−1^, 1313 cm^−1^ and 1585 cm^−1^) bond signals in *C*. *elegans* after 6-h exposure.

To systematically evaluate the fate of QDs through food chain, the Raman signal of both the prey *S. onedensis* (750 cm^−1^, 1128 cm^−1^, 1313 cm^−1^ and 1585 cm^−1^) and the CdSe QDs (202 cm^−1^) were recorded by Raman mapping after exposuring the nematode to the QD-containing bacteria (Fig. 3). After 6-h, the *C. elegans* showed obvious CdSe accumulation in the digestive tracts of  the *C. elegans* was observed, consistent with the fluorescence microscopy results. The co-localization of QDs and cytochrome C, an important membrane-anchored protein in *S. onedensis*, in the gut lumen confirms that the QDs were ingested together with *S. onedensis*. Furthermore, the expansion of cytochrome C signal to the whole nematode body over time revealed a fast assimilation of *S. onedensis* cells by *C. elegans* into subcutaneous tissue. Notably, the distribution of QDs was not fully overlapped with that of cytochrome C signals, indicating that  the CdSe QDs might have been partially transformed to other chemical forms.

### Distribution of Se element in *C. elegans*

**Figure 4:**
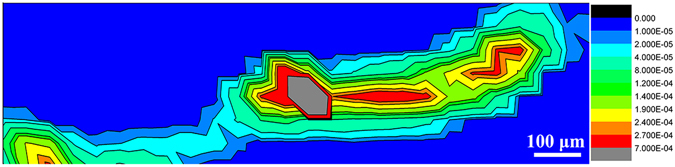
Micro-X-ray fluorescence (*μ*-XRF) spectroscopy of Se element in *C*. *elegans* after 48-h exposure.

The distribution of Se element in *C. elegans* body after 48-h exposure to *S. onedensis* cells was examined by *μ*-XRF spectroscopy. This technique can reduce the possible interference of the biological background compared with other optical and chemical characterization approaches. Figure 4 shows strong Se signal at the digestive tracts, consistent with the fluorescence and Raman results. Notably, the *μ*-XRF spectral signal showed a broader distribution area than the fluorescence and Raman signals in the nematode body, which further supports the transformation of fluorescent CdSe QDs to the other Se species. In contrast, the negative control showed no signals of QDs confirming that the Se was originated from CdSe QDs.

### Biotransformation of CdSe QDs in *C. elegans*

**Figure 5:**
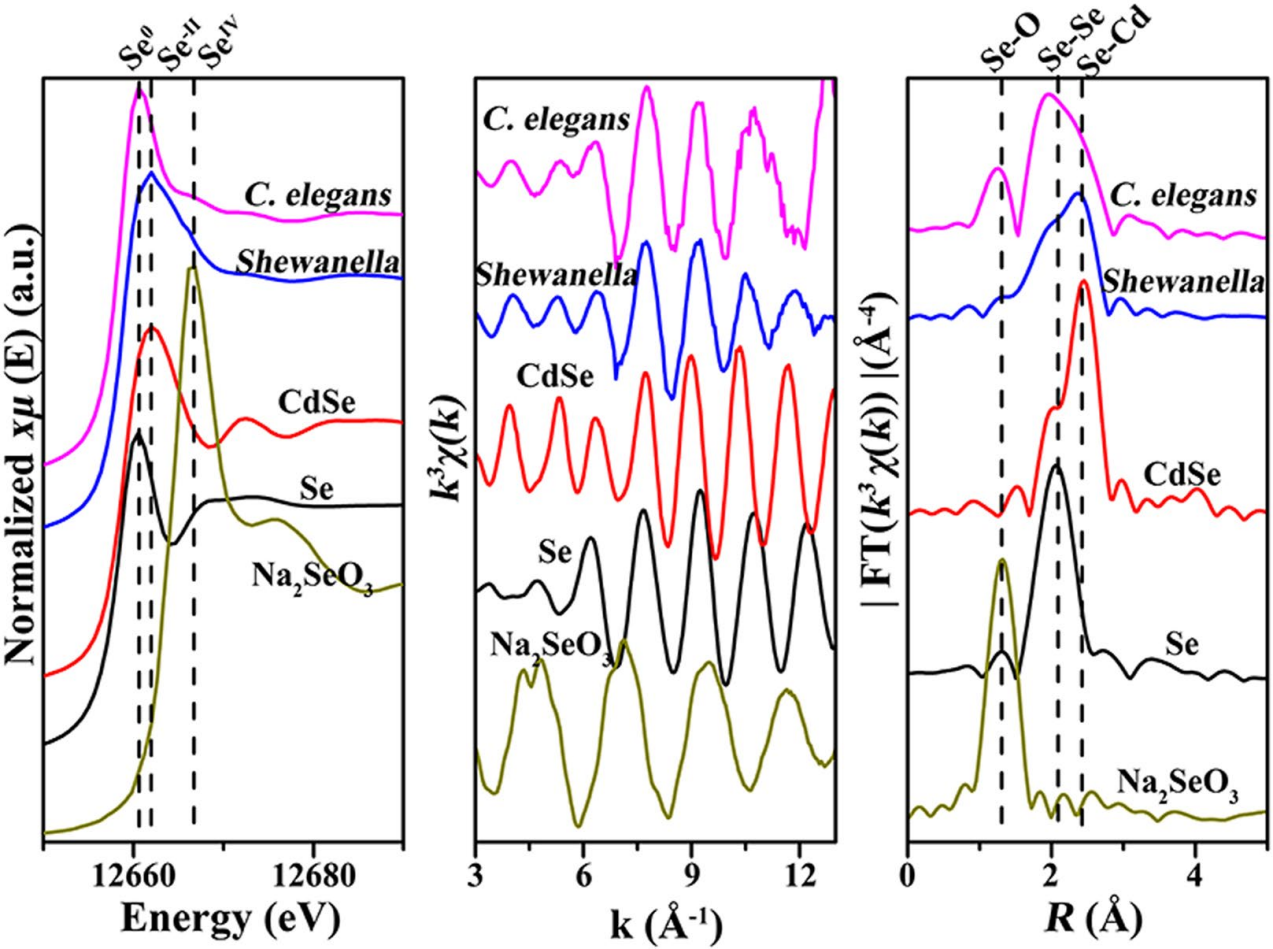
Changes of Se chemical species during food-chain transfer. (**a**) Se *K*-edge normalized XANES spectra of references Se (Se: elemental selenium, Na_2_SeO_3_: sodium selenite and CdSe: cadmium selenide) and biological samples. (**b**) Se *K*-edge EXAFS *k* ^3^ *χ*(*k*) functions and (**c**) Fourier transformed of Se *K*-edge EXAFS *k* ^3^ *χ*(*k*) functions of biological samples and standards.

To shed light on the biotransformation of CdSe QDs in *C. elegans*, Se *K*-edge XAFS was used to examine the chemical composition changes of the QDs during food-chain transfer (Fig. 5). Both X-ray absorption near-edge structure (XANES) and extended X-ray absorption fine structure (EXAFS) spectroscopy were applied for the analysis. The oxidation state of Se can be reflected by the peak positions in XANES (Fig. 5a)^24^. The peak position of QDs-containing *S. onedensis* matched that of CdSe^−II^ references (12,662 eV). Interestingly, after being ingested by *C. elegans*, new peaks emerged at both lower and higher energy positions, matching those of Se^0^ and Na_2_SeO_3_^IV^ standards respectively.

The partial transformation of CdSe QDs to Se^0^ and Na_2_SeO_3_^IV^ was further evidenced by the Fourier transforms of the Se *K*-edge EXAFS (Fig. 5c). Although the QDs-containing *S. onedensis* and *C. elegans* both showed Se-Se and Se-Cd bonds consistent with the Se^0^ and CdSe^−II^ standards, the peak intensities were different. The QDs-containing *S. onedensis* had a higher intensity of Cd-Se bond, while the *C. elegans* showed high intensity of Se-Se bond. In addition, the Se-O bond signal observed in *C. elegans* confirms that the CdSe QDs were partially oxidized to Na_2_SeO_3_^IV^.

## Conclusion

The transformation and distribution of CdSe QDs during their transfer from *S. onedensis* to *C. elegans* through food-chain was systematically examined by using advanced spectrum techniques including fluorescence microscopy, Raman mapping. *μ*-XRF and XAFS spectroscopy. Our results show that the ingested QDs were initially accumulated in the alimentary system, mainly the gut, of *C. elegans*. Later, the distribution area of both the QDs and the cytochrome C increased over time, and partial excretion of the QDs was observed at later stage. Meanwhile, a gradual transformation of CdSe QDs to Se^0^ and Na_2_SeO_3_^IV^ during the food-chain transport was also identified.

## Materials and Methods

### Procedures of QDs transferred from *S. onedensis* to *C. elegans*

The *C. elegans* (Bristol strain N2, wild type) was obtained from Caenorhabditis Genetics Center (Minneapolis, USA) and were grown in Petri dishes on nematode growth medium (17 g/L agar, 2.5 g/L peptone, 3 g/L NaCl, 1 mM CaCl_2_, 1 mM MgSO_4_, 25 mM KPO_4_ buffer and 5 mg/L ethanol). The *Escherichia coli* OP50 strain was used as food source and cultured at 20 °C. The age-synchronized nematode was obtained using hydroxide/hypochlorite treatment according to the description in a previous study^25^ and grown at 20 °C until L4 larval stage. Then, the plates were washed with M9 buffer (3 g/L KH_2_PO_4_, 6 g/L Na_2_HPO_4_, 5 g/L NaCl, 1 mM MgSO_4_) to obtain L4 larval stage worms. The obtained worms were washed three times with M9 buffer to remove excess *E. coli* bacteria. Then transferred them into 200 mL S-complete medium (10 mM potassium citrate, 10 ml trace metals solution, 3 mM CaCl_2_, 3 mM MgSO_4_, 5.85 g/L NaCl, 1 g/L K_2_HPO_4_, 6 g/L KH_2_PO_4_) in 1 L erlenmeyer flask. *S. oneidensis* MR-1 with QDs inside the cells was also added as an extra food source to constitute the food chain. The concentrations of Se and Cd in *S. onedensis* were 101 ± 1.7 and 257 ± 2.0 mg/g protein, respectively. The content of QDs-containing *S. onedensis* fed to *C. elegans* was 280 µg protein/mL. The erlenmeyer flask was put on a shaker with 200 rpm at 20 ^o^C in dark.

### Fluorescence microscopy observation

The fluorescence images of the bacteria and nematodes were obtained using a BX-51 microscope (Olympus Co., Japan) equipped with a 120 W mercury lamp (X-Cite 120 Q). During the observation wideband MWU2 filter (Ex 330-385 nm) and oil immersion objective (100×) were used. DP2-BSW (Olympus Co., Japan) Software was used for the image processing.

### Raman measurements

The Raman spectral analysis and Raman mapping of the cells were conducted using a Thermo Scientific^TM^ DXR^TM^ xi spectrometer equipped with a 532 nm Ar^+^ ion laser and 100× objective. Silicon wafer was used to automatically calibrate the instrument wavelength. In order to improve the signal quality, the samples were placed on a tinfoil plate. The Raman mapping parameters were 0.5 μm diameter spot, 0.3 μm × 0.3 μm aperture and 10 s integration of per Raman spectrum at each location of the laser spot.

### Micro-X-ray fluorescence (*μ*-XRF) spectroscopy analysis

The distribution of Se in *C. elegans* was monitored using *μ*-XRF spectroscopy. Before analysis, the samples were washed three times with 10 mM Tris-HCl (pH 7.6) and immediately fixed with liquid nitrogen on metal-free polyimide film, then frozen dried using vacuum freeze dryer (FreeZone 2.5, Labconco Co., USA). The *μ*-XRF spectroscopy analysis was performed at the 4W1B beamline of Beijing Synchrotron Radiation Facility (BSRF), which can provide 2.5 GeV storage ring and 150 to 250 mA current. The incident X-ray energy was monochromatized by W/B_4_C Double-Multilayer-Monochromator (DMM) at 15 keV and was focused down to 50 μm in diameter by the polycapillary lens. The two-dimensional mapping was acquired by step-mode: the sample was held on a precision motor-driven stage. The Si (Li) solid state detector was used to detect X-ray fluorescence emission lines with live time of 30 s. The data reduction and process were performed using PyMca package^26^.

### X-ray absorption fine structure (XAFS) data collection and analysis

Prior to XAFS analysis, the samples and standards (CdSe, Se) were frozen dried using a vacuum freeze dryer (FreeZone 2.5, Labconco Co., USA). The XAFS analysis was performed at the beamline BL14W1 in SSRF (Shanghai Synchrotron Radiation Facility) with 3.5 GeV storage ring and 300 mA maximum current. The spectra were acquired at room temperature under fluorescence mode using a Si (111) double-crystal monochromator. Before the samples analysis, we first calibrated the energy using Se foil, then the standard ion chamber and Lytle-type detector were used to monitor the samples signal. The acquired XAFS data were background-subtracted, normalized, and Fourier transformed according the standard procedures with the ATHENA program^27, 28^.
